# Artesunate Affects T Antigen Expression and Survival of Virus-Positive Merkel Cell Carcinoma

**DOI:** 10.3390/cancers12040919

**Published:** 2020-04-09

**Authors:** Bhavishya Sarma, Christoph Willmes, Laura Angerer, Christian Adam, Jürgen C. Becker, Thibault Kervarrec, David Schrama, Roland Houben

**Affiliations:** 1 Department of Dermatology, University Hospital Würzburg, 97082 Würzburg, Germany; Sarma_B@ukw.de (B.S.); christoph.willmes@googlemail.com (C.W.); laura-angerer@gmx.de (L.A.); Adam_C@ukw.de (C.A.); Schrama_d@ukw.de (D.S.); 2 Department of Translational Skin Cancer Research, Dermatology, University Hospital Essen, 45147 Essen, Germany; j.becker@dkfz-heidelberg.de; 3 German Cancer Consortium (DKTK), Partner Site Essen and the German Cancer Research Center (DKFZ), 69115 Heidelberg, Germany; 4 Department of Pathology, Centre Hospitalier Universitaire De Tours, INRA UMR 1282 BIP, 37200 Tours, France; thibaultkervarrec@yahoo.fr

**Keywords:** artesunate, Merkel cell carcinoma, MCC, polyomavirus, ferroptosis

## Abstract

Merkel cell carcinoma (MCC) is a rare and highly aggressive skin cancer with frequent viral etiology. Indeed, in about 80% of cases, there is an association with Merkel cell polyomavirus (MCPyV); the expression of viral T antigens is crucial for growth of virus-positive tumor cells. Since artesunate—a drug used to treat malaria—has been reported to possess additional anti-tumor as well as anti-viral activity, we sought to evaluate pre-clinically the effect of artesunate on MCC. We found that artesunate repressed growth and survival of MCPyV-positive MCC cells in vitro. This effect was accompanied by reduced large T antigen (LT) expression. Notably, however, it was even more efficient than shRNA-mediated downregulation of LT expression. Interestingly, in one MCC cell line (WaGa), T antigen knockdown rendered cells less sensitive to artesunate, while for two other MCC cell lines, we could not substantiate such a relation. Mechanistically, artesunate predominantly induces ferroptosis in MCPyV-positive MCC cells since known ferroptosis-inhibitors like DFO, BAF-A1, Fer-1 and β-mercaptoethanol reduced artesunate-induced death. Finally, application of artesunate in xenotransplanted mice demonstrated that growth of established MCC tumors can be significantly suppressed in vivo. In conclusion, our results revealed a highly anti-proliferative effect of the approved and generally well-tolerated anti-malaria compound artesunate on MCPyV-positive MCC cells, suggesting its potential usage for MCC therapy.

## 1. Introduction

Merkel cell carcinoma (MCC) is an aggressive neuroendocrine skin cancer with increasing incidence and mortality rates [1]. The most recent analysis reported 0.7 new cases per 100,000 person-years in 2013 in the United States of America with a predicted 14% increase in cases for 2020 [2], and 0.43 MCC related deaths per 100,000 were reported for 2011 in another study [3]. 95% of the patients with MCC are more than 50 years old, and the tumors commonly develop in chronically sun exposed body areas [4]. Since immunosuppression is a further known risk factor for MCC, an infectious etiology had been suspected [5]. Indeed, in 2008, a human polyomavirus named Merkel cell polyomavirus (MCPyV) was found to be integrated into the genome of Merkel cell carcinoma cells [6], and subsequent studies confirmed that approximately 80% of all MCC cases are associated with MCPyV [7]. Importantly, the integration patterns suggest that clonal expansion of the tumor cells occurs after MCPyV integration sustaining the assumption that viral proteins are causal for tumorigenesis [6,8,9]. Moreover, in MCPyV-positive MCC cells, expression of the viral oncoproteins small and Large T-antigen (sT and LT) can be detected, and these proteins are essential for growth of the tumor cells [10,11] qualifying them as potential therapeutic targets.

The five-year overall survival rate for patients with MCC is only about 40%, although the relative survival rate (compared to an age- and sex-matched population) is 54% [12]. Primary MCCs are excised by surgery, and adjuvant radiotherapy of the primary tumor location and the lymph node region is recommended [13]. Until recently, the metastatic disease was treated preferentially with various, not-standardized chemotherapeutic regimens, all of which could not improve survival of the patients significantly [14]. Recently, however, antibodies targeting the immune suppressive protein programmed cell death protein 1 (PD-1) or its ligand PD-L1 have demonstrated high response rates of 56 in first-line and 32% in second-line treatment, respectively for patients with stage IV disease [15,16]. Indeed, the PD-L1 targeting antibody Avelumab was the first treatment for metastatic MCC approved both in the US and European Union [17]. Importantly, data available so far suggest that responses of MCC patients to checkpoint inhibition are frequently long-lasting [18,19]. However, despite this encouraging progress, many patients do not respond and a substantial number of patients develop early secondary resistance [18,20]. Therefore, there is strong need for therapeutic approaches for patients’ refractory to immune checkpoint inhibition. Furthermore, in developing countries there is a particular need for alternative MCC treatment options, since the high costs of checkpoint antibodies may limit their usage [21]

Artesunate is a semi-synthetic derivative of artemisinin, the active ingredient of the traditional Chinese medicinal herb *Artemisia annua* [22]. Artesunate is applied as first-line drug for the treatment of malaria which is caused by an infection with protozoa of the genus *Plasmodium* [23]. Although artesunate represents the most effective and safe anti-malarial drug [24,25], its mode of action is only incompletely understood [26]. Interestingly, artesunate has also been demonstrated to be specifically cytotoxic to cancer cells from several tumor entities [27,28]. This cytotoxicity was ascribed to artesunate impacting a multitude of signaling pathways and cell death modes [22]. For the latter, induction of apoptosis [29,30,31] or ferroptotic cell death [32,33,34] have been reported most frequently. Importantly, besides these anti-cancer effects, it also exerts anti-viral activities towards a broad range of viruses [35,36]. Therefore, we examined whether MCPyV-associated MCC cells are sensitive to this compound.

Here we demonstrate that artesunate effectively induces cell death of MCPyV-positive MCC cells in vitro mainly through ferroptosis, while apoptosis appears not to be involved. Moreover, in a mouse model, we demonstrate that artesunate can be applied to inhibit MCC tumor growth *in vivo.*

## 2. Results

### 2.1. Artesunate Effectively Inhibits Growth of MCPyV-Positive MCC Cell Lines In Vitro

Artesunate has been shown to mediate both anti-viral and anti-tumor activity [28,36]. Due to the viral carcinogenesis of most MCCs, we tested in an initial experiment, the effect of artesunate on a panel of MCPyV-positive classical MCC cell lines and some non-classical MCPyV-negative MCC cell lines. Melanoma cell lines and primary fibroblasts were included as further controls. The drug was used at concentrations of 1 and 10 µM and its effect on cell growth and metabolism was determined by the MTS assay. While growth and survival of primary fibroblasts and melanoma cell lines was largely unaffected at the given concentration, in particular the MTS signals of the MCPyV-positive MCC cell lines WaGa and MKL-1 were largely reduced (Supplementary Figure S1).

### 2.2. Reduced Large T Antigen Expression in Response to Artesunate

**Figure 1:**
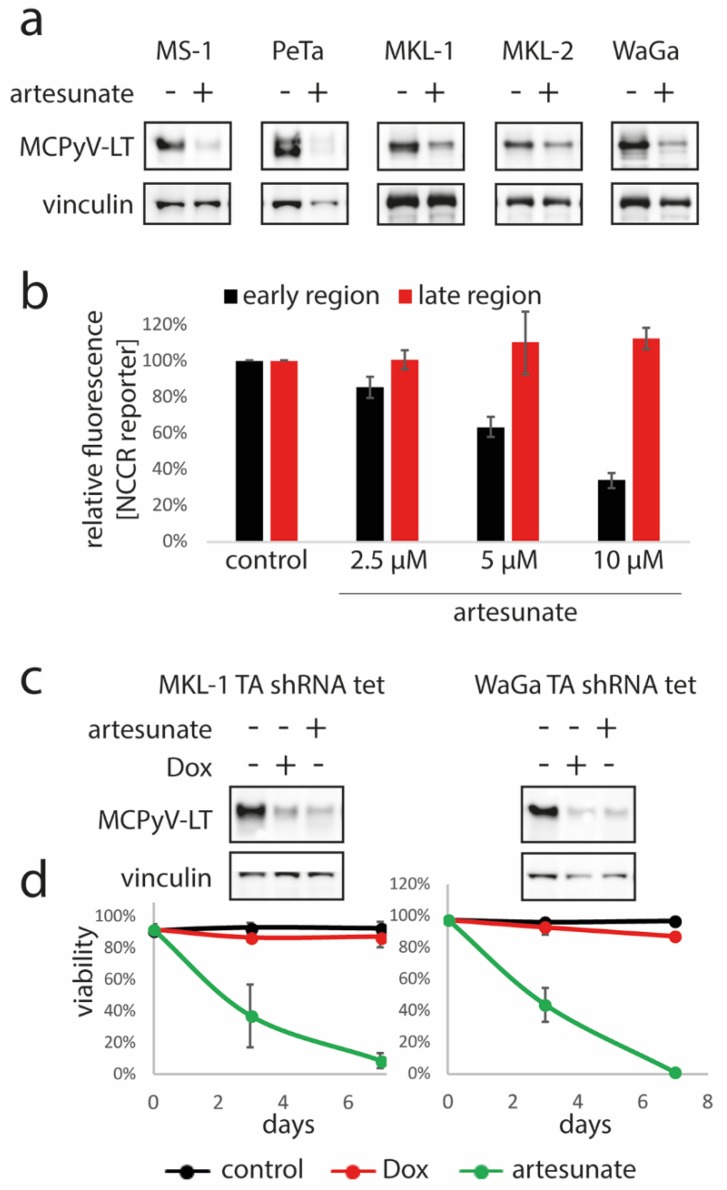
Artesunate-induced repression of MCPyV-LT expression in MCC cells is not crucial for its cytotoxic effects. (**a**) The indicated MCPyV-positive Merkel cell carcinoma (MCC) cell lines were incubated for three days in the absence or presence of artesunate (10 µM for MKL-1, MKL-2 and WaGa and 12.5 µM for MS-1 and PeTa) followed by immunoblot analysis. (**b**) MKL-1 cells stably transduced with a bi-directional non-coding control region (NCCR) reporter construct were treated for five days with the indicated artesunate concentrations followed by flow cytometric analysis. Mean fluorescence for early and late region were recorded, and mean values (± SD) are displayed. (**c**,**d**) MKL-1 and WaGa cells stably transduced with a vector allowing doxycyclin (Dox)-inducible expression of an shRNA targeting MCPyV TA were treated either with Dox (1 µM) or artesunate (10 µM) for 7 days, respectively. (**c**) large T antigen (LT) expression was analyzed by immunoblot. (**d**) Trypan blue exclusion assay was applied to determine viability in the course of time. Mean values (± SD) of at least four independent experiments are depicted.

MCPyV-positive MCC cell lines depend on expression of the viral T antigens and in particular LT for growth [37,38]. Therefore, seeking for a potentially virus-related mechanism of growth inhibition induced by artesunate, we analyzed whether it affects LT expression. Indeed, in all five analyzed MCC cell lines, immunoblot analysis revealed decreased LT protein expression upon a three-day incubation with artesunate (Figure 1a; Supplementary Figure S2a).

To investigate whether artesunate affects the promoter driving T antigen expression, we made use of a reporter construct in which the bi-directional MCPyV non-coding control region (NCCR) controls expression of a green and a red fluorescent protein representing the early and late region, respectively. Indeed, MKL-1 cells transduced with the reporter demonstrated a dose dependent reduction of green fluorescence upon treatment with artesunate, while red fluorescence was not affected (Figure 1b; Supplementary Figure S3) suggesting that artesunate may specifically downregulate LT via repression of its NCCR-dependent transcription.

### 2.3. Artesunate Exerts Stronger Cytotoxic Effects on MCC Cells than TA Knockdown

Next, we asked whether the inhibition of T antigen (TA) expression could be a crucial mediator of the artesunate-induced effects on MCC cells. To answer this question, we compared loss of viability following artesunate treatment with cell death induced upon shRNA-mediated TA knockdown. To this end, MKL-1 and WaGa cells transduced with a lentiviral vector allowing doxycyclin-inducible expression of an shRNA targeting both T antigens were used. Addition of doxycyclin to these cells led to an efficient knockdown evident by reduced LT in immunoblot analysis (Figure 1c; Supplementary Figure S2b), which however was associated with only a minor increase in dead cells as assessed by the trypan blue exclusion assay (Figure 1d). In contrast, incubation with 10 µM artesunate, which was associated with a similar level of LT reduction (Figure 1c; Supplementary Figure S2b), induced massive cell death within 7 days. These results argue against repression of TA expression being the sole mechanism for the observed artesunate-mediated cytotoxicity on MCPyV-positive MCC cell lines.

### 2.4. Expression of the T Antigens Sensitizes the MCPyV-Positive Cell Line WaGa to Artesunate

**Figure 2:**
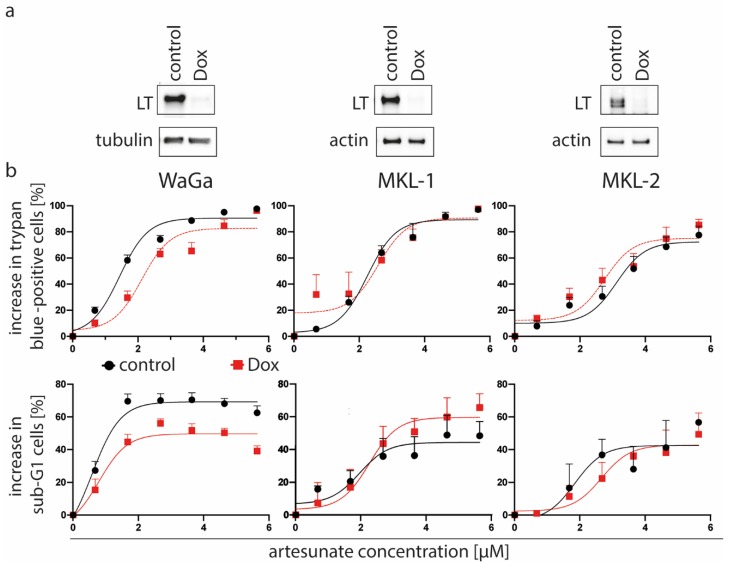
T antigen knockdown is associated with decreased artesunate sensitivity of WaGa but not MKL-1 and MKL-2 cells. We used the indicated cell lines which were stably transduced with a vector allowing doxycyclin (Dox)-inducible expression of a T antigen (TA) shRNA as well as with a vector constitutively expressing an RB1 shRNA. (**a**) Following 5 days in the presence or absence of Dox (1 µM) TA knockdown was evaluated by immunoblot analysis. (**b**) Then artesunate dose-response curves were recorded for control and Dox-treated cells applying the trypan blue exclusion assay as well as determination of the Sub-G1 population following propidium iodide staining of fixed cells. Displayed are mean values (+ SE) of at least three independent experiments.

As the results so far did not exclude a possible role of MCPyV for the artesunate-induced cytotoxicity, we analyzed next whether TA knockdown in MCPyV-positive MCC cells may affect their artesunate sensitivity. Since many cytotoxic drugs are less effective against non-proliferating cells [39], we used MKL-1, MKL-2 and WaGa cells which in addition to the inducible TA shRNA, constitutively express a Retinoblastoma protein 1 (RB1) shRNA rescuing the growth arrest induced by LT knockdown [38]. Control cells without doxycycline treatment and cells incubated for four days with doxycycline to repress T-antigen expression (Figure 2a) were then treated with artesunate ranging from 1.6 to 50 µM. Two assays, namely the trypan blue dye exclusion assay and DNA staining using propidium iodide were used to analyze cell viability of both groups. Interestingly, both assays demonstrated that WaGa cells with repressed T antigen showed increased cell viability upon artesunate treatment compared to the respective controls without the knockdown (Figure 2b). Therefore, T antigen expression seems to sensitize WaGa cells to artesunate induced cell death. For MKL-1 and MKL-2 cells, however, T antigen knockdown did not alter their sensitivity towards artesunate (Figure 2b).

In addition, artesunate induced cell death was preceded by a G2/M arrest (Supplementary Figure S4), while TA knockdown has been demonstrated to cause an arrest in G1 [11], further sustaining the conclusion that artesunate has important impacts on MCC cells in addition to T antigen repression.

### 2.5. No Signs of Apoptotic Cell Death Are Induced by Artesunate in Most MCPyV-Positive MCC Cell Lines

**Figure 3:**
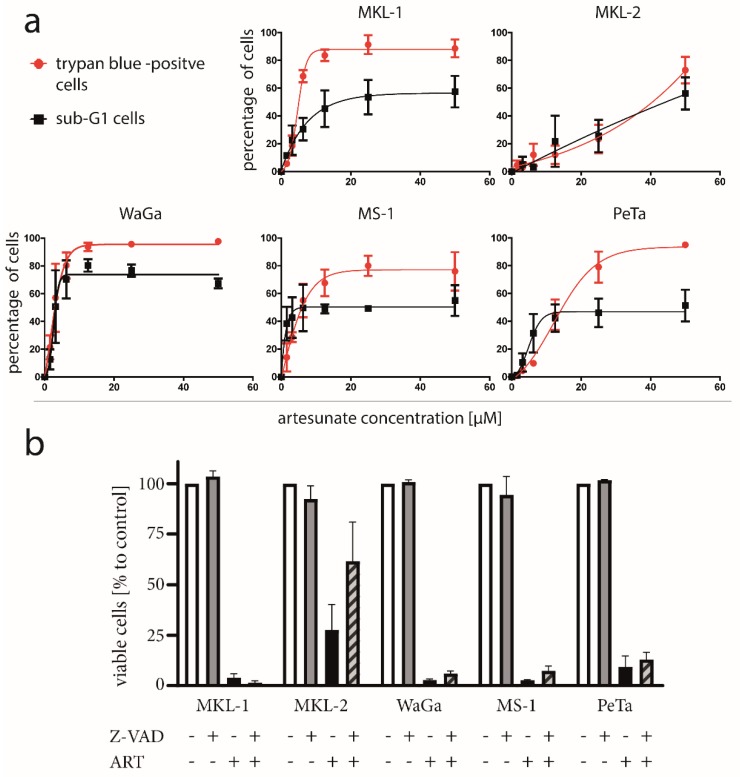
No signs of apoptotic cell death in most artesunate-treated MCC cells. (**a**) The indicated cell lines were treated for three days with increasing concentrations of artesunate. Then cell death was measured by the trypan blue exclusion assay. Additionally, cells were fixed and stained with propidium iodide to determine the increase in cells with a DNA content of less than 2N (sub-G1) (**b**) Cells were treated with 50 µM artesunate (ART) in the presence and absence of 20 µM of the caspase inhibitor Z-VAD. Viability was assessed by the trypan blue exclusion assay. Statistical testing applying ANOVA did not reveal significant differences.

To further scrutinize artesunate’s cytotoxicity towards MCPyV-positive MCC cells, we recorded dose response curves for five MCC cell lines applying two different cell death assays. Interestingly, we observed for four of the five cell lines, a significant difference between cell death induction as assayed by trypan blue exclusion compared to the appearance of a sub-G1 population in particular at higher artesunate concentrations (Figure 3a; Supplementary Figure S5). Indeed, cells with DNA less than 2N were less frequent than cells that had lost membrane integrity. This suggests that artesunate-induced death is not preceded by DNA fragmentation, a well-known characteristic of apoptosis [40]. Hence, apoptosis, a frequently described result of artesunate treatment in cancer cells [29,30,31,41], seems not to represent a crucial mechanism in these MCPyV-positive MCC cell lines. Only for MKL-2, no difference could be observed between the two dose response curves suggesting a possible contribution of apoptotic cell death.

To further evaluate these findings, we applied the pan caspase inhibitor benzyloxycarbonyl-ValAla-Asp (OMe) fluoromethylketone (Z-VAD-FMK), which bears the capability to suppress caspase-dependent apoptosis [42]. Although for MKL-2 an increase of viable cells in the presence of Z-VAD-FMK was observed, a significant rescue from artesunate induced cell death could not be detected for any of the five MCC cell lines (Figure 3b; differences tested with ANOVA and subsequent post hoc tests comparing values to those of artesunate-treated cells).

Finally, we investigated morphologic changes associated with artesunate treatment of MCPyV-positive MCC cell lines since apoptosis is characterized by characteristic features like cell shrinkage, membrane blebbing and formation of apoptotic bodies [43,44]. However, none of these characteristics were detectable when we analyzed the two non-spheroidal cell lines WaGa and PeTa by time lapse microscopy. Indeed, upon artesunate treatment, the opposite of shrinkage, i.e., cell swelling, was observed before death occurred (Supplementary Figure S6).

In conclusion, several observations suggest that at least in most artesunate-treated MCPyV-positive cell lines, apoptosis is not induced, and the morphologic feature of cell swelling hints to either necroptosis or ferroptosis provoked by artesunate [44,45].

### 2.6. Ferroptosis as a Key Player in Artesunate-Induced Cytotoxicity in MCPyV-Positive Cells

**Figure 4:**
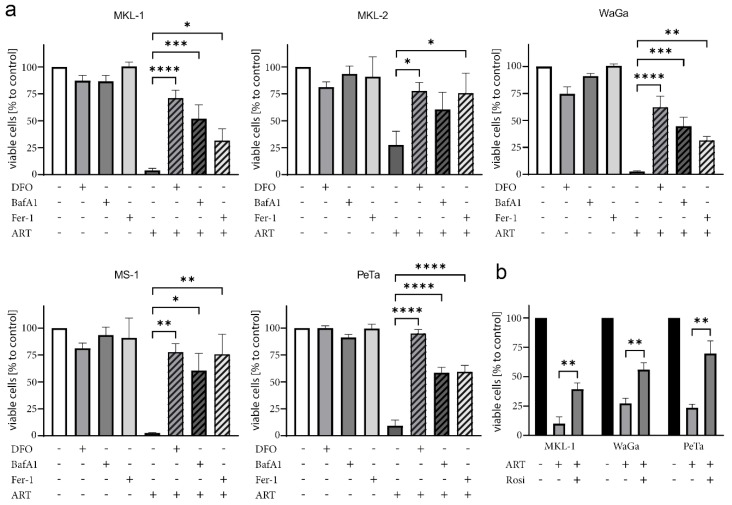
Ferroptosis inhibitors rescue MCPyV-positive MCC cells from artesunate-induced cell death. The indicated MCPyV-positive MCC cell lines were cultured in the absence or presence of 50 µM artesunate (ART). Additionally, either 10 µM of the radical-trapping antioxidant ferrostatin-1 (Fer-1), 100 µM of the iron-chelator deferoxamine (DFO), 50 nM of the autophagy inhibitor bafilomycin-A1 (BAF-A1) (**a**) or 25 µM of the ACSL4 inhibitor rosiglitazone (Rosi) (**b**) were included in the culture medium. After two days of co-treatment, viability was assessed by the trypan blue exclusion assay. Mean values (± SD) of at least three independent experiments are displayed. The effect of multiple treatment and inhibitor combinations was tested by ANOVA followed by post-hoc test comparing the effect always against the one observed for artesunate treatment. (* *p* < 0.05; ** *p* < 0.01; *** *p* < 0.001; **** *p* < 0.0001).

Previous studies had revealed the capability of artesunate to induce ferroptosis, an iron-dependent cell death mode characterized by lipid peroxidation [32,33,34]. Therefore, we next applied several specific inhibitors to test for ferroptotic features of artesunate-treated MCC cells. In this regard, rescue from cell death by the radical-trapping antioxidant ferrostatin-1 (Fer-1) which blocks lipid peroxidation [46] is regarded as one of the features defining ferroptosis [47]. Indeed, in all investigated MCC cell lines artesunate-induced cell death was significantly reduced by Fer-1. In addition, inhibition of artesunate-triggered viability loss by the iron-chelator deferoxamine (DFO) confirmed a ferroptotic process (Figure 4a).

Furthermore, the effect of the vacuolar ATPase inhibitor bafilomycin-A1 (BAF-A1) in combination with artesunate was investigated. Multifaceted outcomes, like apoptosis induction or inhibition of autophagy, have been described for BAF-A1 [48,49]. However, BAF-A1 has also been observed to suppress ferroptosis, giving rise to one of the arguments linking autophagy to the ferroptotic process [47,50,51]. Such a link appears to exist also in MCC cell lines since among the tested inhibitors, BAF-A1 most efficiently suppressed artesunate-induced cell death in the MCPyV-positive MCC cell lines (Figure 4a).

A further reported step essential for ferroptosis is the inhibition of cystine import, which is necessary for antioxidant production [52,53]. In line with the notion that artesunate-induced cell death requires reduced cystine import, β-mercaptoethanol, which promotes cystine uptake [54], repressed cell death in artesunate-treated MCC cells (Supplementary Figure S7).

Finally, we tested rosiglitazone (Rosi), an inhibitor of the Acyl-CoA synthetase long-chain family member 4 (ACSL4). This enzyme has been demonstrated to be involved in ferroptosis execution by converting long-chain poly-unsaturated fatty acids (PUFAs) to their corresponding fatty acyl-CoA variants [55,56]. Indeed, Rosi exerted a protective effect on all three tested artesunate-treated MCC cell lines (Figure 4b).

These results suggest that artesunate kills MCPyV-positive MCC cells by dysregulating lipid metabolism and autophagy resulting in ferroptosis.

### 2.7. Artesunate Inhibits Tumor Growth In Vivo

**Figure 5:**
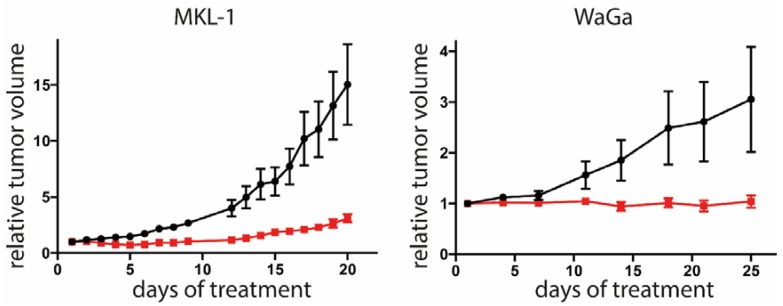
Tumor growth is restricted in artesunate-treated mice. Immunodeficient NOD/Scid mice received subcutaneous injection of either MKL-1 or WaGa cells. When tumors reached a size of 100 mm^3^, the mice were randomly assigned to control group (n = 6 for WaGa and n = 5 for MKL-1, since in one animal no tumor growth was observed) or treatment group (n = 6). Each mouse from the treatment group was subjected to daily intraperitoneal injections with 100 mg/kg artesunate. The control group received injection of an equal volume of solvent (2% DMSO in PBS). The experiment was terminated once individual tumors of the control group reached the maximum tolerable size. Depicted are the means (± SEM). Statistical analyses of area under the curves for the two models were *p* < 0.001 for MKL-1 and 0.0305 for WaGa (unpaired *t*-test).

To evaluate whether artesunate can affect growth of MCPyV-positive tumors in a living organism, we used xenotransplantation mouse models based on subcutaneous transplantation of the cell lines MKL-1 or WaGa [57]. Following injection of the tumor cells, the animals were monitored until they developed visible and palpable tumors measuring approximately 150 mm^3^. Subsequently, 100 mg/kg body weight artesunate was administered intraperitoneally while control mice received the same volume of vehicle control. Artesunate treatment significantly reduced tumor growth of both MKL-1 and WaGa tumors (Figure 5).

## 3. Discussion

The term drug repositioning (also called drug repurposing) describes the use of established drugs for new therapeutic purposes. Drug repositioning is a well-established process approved by regulatory agencies that allows fast identification of new treatment options, usually associated with less costs and lower risks for patients compared to the development of new drugs [58]. While some compounds (e.g., thalidomide, zoledronic acid, celecoxib) have already been successfully repositioned for cancer treatment, other drugs like, e.g., artesunate are currently in the process for possible repositioning [58].

Artesunate is a derivative of artemisinin, an extract from the plant *Artemisia annua* Linne [22]. Notably, the discovery that artemisinin-class substances can be applied as potent therapeutics for malaria patients, was awarded with the Nobel Prize in 2015 [59]. Indeed, artesunate exerts superior antimalarial effects in clinical application and is characterized by an excellent safety profile [60]. Furthermore, in recent years, several additional activities beyond anti-malarial activity have been observed [22,61]. In this respect, pre-clinical studies on artesunate have demonstrated anti-tumor activity against many different cancers including colon caancer [29], lung adenocarcinoma [31], pancreatic cancer [33], breast cancer [62] and different hematological malignancies [30,32,63]. The present study adds MCPyV-positive MCC to this list as we demonstrate the capability of artesunate to restrict growth of virus-positive MCC cells in vitro as well as in xenotransplantation mouse models *in vivo*.

The question whether presence of the viral proteins in these cells affects their artesunate sensitivity could not be fully answered. In line with different reported anti-viral effects of artesunate [35,36] including impairment of the polyomavirus life cycle [64,65], we observed repression of T antigen expression in artesunate-treated MCPyV-positive MCC cells. This was different compared to human papilloma virus infected cervical cancer cells in which expression of the viral oncogenes was not affected by the related compound dihydroartemisinin [66]. However, although T antigens are essential for growth of MCPyV-positive MCC cells [11], the cytotoxicity of artesunate towards these cells seems not to depend on viral-protein repression. Indeed, in this respect, artesunate was more potent than T antigen knockdown. It was only in one MCC cell line (WaGa), that sensitivity towards artesunate was reduced upon knockdown of T antigen expression.

Irrespective of a possible contribution of the T antigens to artesunate-induced cell death of MCPyV-positive MCC cells, a set of inhibitor experiments suggest that artesunate induces ferroptosis and not apoptosis in these cells. Among the multitude of different modes of regulated cell death, an important distinction is their dependence on caspases. In this regard, apoptosis and pyroptosis require activation of these proteases while necroptosis, ferroptosis, parthanatos, alkaliptosis and oxeiptosis are caspase-independent [45]. In the case of artesunate-treated MCPyV-positive MCC cells, the pan caspase inhibitor Z-VAD-FMK did not significantly reduce cell death. In contrast, inhibitors targeting different steps of the ferroptotic pathway were effective in rescuing artesunate-triggered killing of virtually all five investigated MCC cell lines.

Ferroptosis is a mode of programmed cell death that is characterized by an iron-dependent accumulation of lipid peroxides [48]. Interestingly, ferroptosis is considered to be pro-inflammatory and immunogenic, due to release of damage-associated molecular patterns (DAMPs) [67,68]. Hence, besides the direct effects on the tumor cells, artesunate may also support anti-tumor immune responses. However, direct evidence for this possibility is still scarce and further investigations on this topic are necessary [67]. Notwithstanding, following preclinical evaluation of the anti-tumoral activity of artesunate in recent years, we have now reached a phase of human trials for the treatment of cancer patients with artesunate. In this respect, several phase 1 and phase two studies (colorectal cancer, hepatocellular carcinoma, breast cancer and several intraepithelial neoplasias) are ongoing (www.clinicaltrials.gov), and for a few trials results have already been published. These reports highlighted the favorable tolerability of artesunate [69,70,71,72], and some even found first hints for clinical activity [69,70]. It may be interesting to see how artesunate, maybe even in combination with immune checkpoint inhibitors, performs in cancer trials in the future. Certainly, MCPyV-positive MCC patients may be included in such studies.

## 4. Materials and Methods

### 4.1. Ethics Statement

Animal experiments were performed according to the legal requirements and approved by the Regierung von Unterfranken (RUF 55.2.2 -22532.2 -925-18).

### 4.2. Cloning and Usage of an NCCR Reporter Construct

To allow assessment of the transcriptional activity of the MCPyV noncoding control region (NCCR) by flow cytometry, we cloned a lentiviral reporter construct in which we placed a green and a red fluorescent protein 3′ and 5′ of the NCCR (Supplementary Figure S8). We included in addition to the mere NCCR, also the sequences coding for the N-terminus of sT and VP2 in the construct to prevent losing potential regulatory elements extending into the respective coding region. To this end, mNeongreen and mCherry coding sequences were cloned in frame with the first 78 codons of sT and the first 64 codons of VP2, respectively (Supplementary Figure S8a). This cassette was inserted into the multiple cloning site of pLVX-Puro (Clontech) yielding the construct pLVX NCCR mNeongreen mRuby3 (the map is available upon request).

Lentivirus particles were generated as described [39] and used for infection of MKL-1 cells. Red and green fluorescence was analyzed on a CytoFLEX flow cytometer (Beckman Coulter).

### 4.3. Cell Culture

MCPyV-positive MCC cell lines MKL-1 [73], MKL-2 [74], MS-1 [75], WaGa and PeTa (both described in [9]) were cultivated in RPMI-1640 medium (Sigma Aldrich) supplemented with 10% FBS (Biochrom GmbH), 100 U/mL penicillin and 0.1 mg/mL streptomycin (Sigma Aldrich).

MKL-1, MKL-2 and WaGa cells with constitutive expression of an shRNA targeting RB1 and doxycyclin-inducible expression of an shRNA targeting both T antigens (TA shRNA tet), have been described previously [38].

### 4.4. Immunoblotting

Cells were lysed using the ELB lysis buffer containing 150 mM NaCl, 50 mM Hepes pH 7.5, 5 mM EDTA, 0.1% NP-40, 20 mM β-glycerophosphate, 0.5 mM sodium orthovanadate and a protease inhibitor (Roche). The immunoblotting procedure was performed as described [38]. The antibodies used in this study were directed against MCPyV-LT (CM2B4; Santa Cruz Biotechnologies), β-tubulin (TUB 2.1; Sigma-Aldrich, Ottobrunn, Germany) and vinculin (hVIN-1; Sigma-Aldrich). Uncropped blots are given in Supplementary Figures S9 and S10)

### 4.5. MTS Assay

Cell lines were seeded in sextuplicate per condition in 96-well plates. Following 5 days of incubation with 0. 1 and 10 µM artesunate (Sigma Aldrich), the MTS proliferation assay (Promega, Mannheim, Germany) was applied according to the manufacturer’s instructions.

### 4.6. DNA Staining

Cells were fixed with ice-cold 90% ethanol followed by a one-hour treatment with propidium iodide mix (PBS + 1% FCS + 0.1 mg/mL propidium iodide + 0.1 mg/mL RNAse A). Analysis was then performed by flow cytometry.

### 4.7. Trypan Blue Exclusion Assay

Cells were stained with 0.4% trypan blue in PBS (Sigma Aldrich), and the number of living, dye-excluding cells as well as the dead blue-stained cells were counted using a hemocytometer.

### 4.8. Time Lapse Microscopy

WaGa and PeTa cells were seeded at a density of 1 × 10^5^ cells per well in µ-Slides (Ibidi) and treated with 50 μM artesunate. Morphologic changes in the course of time were recorded using a Nikon Eclipse Ti microscope.

### 4.9. Animal Experiments

Five-week-old female NOD.CB17/*Prkdc*scid mice (Charles River) were used for the xenotransplantation experiments. They were housed under specific pathogen-free conditions. Each mouse was injected subcutaneously with a suspension of 5 × 10^6^ MKL-1 or WaGa tumor cells mixed with an equal volume of Matrigel (Corning) in a total volume of 100 μL. The tumor size was measured daily using a vernier calipers and the volume was calculated using the formula (V = π/6 × a^2^ × b (a: length; b: height). Once the tumor size reached approximately 150 mm^3^, the mice were divided into the control group (n = 6 for WaGa and n = 5 for MKL-1, since in one animal, no tumor growth was observed) and treatment group (n = 6). Each mouse from the treatment group was subjected to daily intraperitoneal injections with 100 mg/kg of artesunate, which was dissolved in DMSO and then brought to a total volume of 200 μL with PBS prior to injection. Similarly, the control group was injected with the same volume of DMSO in 200 μL of PBS (2% DMSO). The experiments were terminated once the tumors of the control group reached the maximum tolerable size.

### 4.10. Statistical Analysis

Statistical analyses were completed with Prism 5.03 (GraphPad Software, Inc;. San Diego, CA 92108, USA). Since cell volume distribution did not pass normality test; the volumes were compared by non-parametric Mann–Whitney test. The effect of multiple treatment and inhibitor combinations was tested by ANOVA followed by post-hoc test comparing the effect always against ones observed for artesunate treatment. Adjusted p values following Dunnett multiple comparison testing were given. For tumor growth curves, first area under the curve of tumor sizes (baseline set to zero) normalized to the size at the start of treatment, were determined. These values were compared by unpaired *t*-test.

## 5. Conclusions

Artesunate induced ferroptosis in MCPyV-positive MCC cells in vitro and restricted growth of MCC xenograft tumors in vivo. These results suggest that the established antimalarial therapeutic may be applied to treat patients with MCPyV-positive MCC.

## Supplementary Materials

The following are available online at https://www.mdpi.com/2072-6694/12/4/919/s1, Figure S1: tMCC cell lines are more sensitive towards artesunate than melanoma cell lines or primary fibroblasts, Figure S2: Densitometric analyses of all immunoblots presented in the publication, Figure S3: Artesunate represses NCCR driven early region transcription (representative histograms of the NCCR Reporter-Assay corresponding to the bar graph in Figure 1b), Figure S4: Artesunate induces G2/M arrest in MCPyV-positive MCC cells, Figure S5: Propidium iodide staining of artesunate treated MCPyV-positive MCC cells (representative histograms corresponding to Figure 3a), Figure S6: Artesunate induces cell swelling of MCPyV-positive MCC cells, Figure S7: β-Mercaptoethanol, an activator of cystine uptake, represses artesunate-induced cell death, Figure S8: An MCPyV NCCR reporter construct, Figure S9: Uncropped blots from Figure 1a,c, Figure S10: Uncropped blots from Figure 2.

## References

[1] Hodgson N.C. (2005). Merkel cell carcinoma: Changing incidence trends. J. Surg. Oncol..

[2] Paulson K.G., Park S.Y., Vandeven N.A., Lachance K., Thomas H., Chapuis A.G., Harms K.L., Thompson J.A., Bhatia S., Stang A. (2017). Merkel Cell Carcinoma: Current United States Incidence and Projected Increases based on Changing Demographics. J. Am. Acad. Derm..

[3] Fitzgerald T.L., Dennis S., Kachare S.D., Vohra N.A., Wong J.H., Zervos E.E. (2015). Dramatic Increase in the Incidence and Mortality from Merkel Cell Carcinoma in the United States. Am. Surg..

[4] Miller R.W., Rabkin C.S. (1999). Merkel cell carcinoma and melanoma: Etiological similarities and differences. Cancer Epidemiol. Biomark. Prev..

[5] Buell J.F., Trofe J., Hanaway M.J., Beebe T.M., Gross T.G., Alloway R.R., First M.R., Woodle E.S. (2002). Immunosuppression and merkel cell cancer. Transplant. Proc..

[6] Feng H., Shuda M., Chang Y., Moore P.S. (2008). Clonal Integration of a Polyomavirus in Human Merkel Cell Carcinoma. Science.

[7] Tothill R., Estall V., Rischin D. (2015). Merkel cell carcinoma: Emerging biology, current approaches, and future directions. Am. Soc. Clin. Oncol. Educ. Book. Am. Soc. Clin. Oncol. Meet..

[8] Sastre-Garau X., Peter M., Avril M.F., Laude H., Couturier J., Rozenberg F., Almeida A., Boitier F., Carlotti A., Couturaud B. (2009). Merkel cell carcinoma of the skin: Pathological and molecular evidence for a causative role of MCV in oncogenesis. J. Pathol..

[9] Schrama D., Sarosi E.M., Adam C., Ritter C., Kaemmerer U., Klopocki E., Konig E.M., Utikal J., Becker J.C., Houben R. (2019). Characterization of six Merkel cell polyomavirus-positive Merkel cell carcinoma cell lines: Integration pattern suggest that large T antigen truncating events occur before or during integration. Int. J. Cancer J. Int. Du Cancer.

[10] Shuda M., Kwun H.J., Feng H., Chang Y., Moore P.S. (2011). Human Merkel cell polyomavirus small T antigen is an oncoprotein targeting the 4E-BP1 translation regulator. J. Clin. Investig..

[11] Houben R., Shuda M., Weinkam R., Schrama D., Feng H., Chang Y., Moore P.S., Becker J.C. (2010). Merkel cell polyomavirus-infected Merkel cell carcinoma cells require expression of viral T antigens. J. Virol..

[12] Lemos B.D., Storer B.E., Iyer J.G., Phillips J.L., Bichakjian C.K., Fang L.C., Johnson T.M., Liegeois-Kwon N.J., Otley C.C., Paulson K.G. (2010). Pathologic nodal evaluation improves prognostic accuracy in Merkel cell carcinoma: Analysis of 5823 cases as the basis of the first consensus staging system. J. Am. Acad. Derm..

[13] Poulsen M. (2005). Merkel cell carcinoma of skin: Diagnosis and management strategies. Drugs Aging.

[14] Eng T.Y., Boersma M.G., Fuller C.D., Goytia V., Jones W.E., Joyner M., Nguyen D.D. (2007). A comprehensive review of the treatment of Merkel cell carcinoma. Am. J. Clin. Oncol..

[15] Nghiem P.T., Bhatia S., Lipson E.J., Kudchadkar R.R., Miller N.J., Annamalai L., Berry S., Chartash E.K., Daud A., Fling S.P. (2016). PD-1 Blockade with Pembrolizumab in Advanced Merkel-Cell Carcinoma. N. Engl. J. Med..

[16] Kaufman H.L., Russell J., Hamid O., Bhatia S., Terheyden P., D’Angelo S.P., Shih K.C., Lebbe C., Linette G.P., Milella M. (2016). Avelumab in patients with chemotherapy-refractory metastatic Merkel cell carcinoma: A multicentre, single-group, open-label, phase 2 trial. Lancet Oncol..

[17] Kim E.S. (2017). Avelumab: First Global Approval. Drugs.

[18] Nghiem P., Bhatia S., Lipson E.J., Sharfman W.H., Kudchadkar R.R., Brohl A.S., Friedlander P.A., Daud A., Kluger H.M., Reddy S.A. (2019). Durable Tumor Regression and Overall Survival in Patients With Advanced Merkel Cell Carcinoma Receiving Pembrolizumab as First-Line Therapy. J. Clin. Oncol. Off. J. Am. Soc. Clin. Oncol..

[19] D’Angelo S.P., Russell J., Lebbé C., Chmielowski B., Gambichler T., Grob J.-J., Kiecker F., Rabinowits G., Terheyden P., Zwiener I. (2018). Efficacy and Safety of First-line Avelumab Treatment in Patients With Stage IV Metastatic Merkel Cell Carcinoma: A Preplanned Interim Analysis of a Clinical Trial. JAMA Oncol..

[20] Kaufman H.L., Russell J.S., Hamid O., Bhatia S., Terheyden P., D’Angelo S.P., Shih K.C., Lebbé C., Milella M., Brownell I. (2018). Updated efficacy of avelumab in patients with previously treated metastatic Merkel cell carcinoma after ≥1 year of follow-up: JAVELIN Merkel 200, a phase 2 clinical trial. J. Immunother. Cancer.

[21] Marin-Acevedo J.A., Dholaria B., Soyano A.E., Knutson K.L., Chumsri S., Lou Y. (2018). Next generation of immune checkpoint therapy in cancer: New developments and challenges. J. Hematol. Oncol..

[22] Efferth T. (2017). From ancient herb to modern drug: Artemisia annua and artemisinin for cancer therapy. Semin Cancer Biol..

[23] Burrows J.N., Chibale K., Wells T.N. (2011). The state of the art in anti-malarial drug discovery and development. Curr. Top. Med. Chem..

[24] Wang J., Zhang C.J., Chia W.N., Loh C.C., Li Z., Lee Y.M., He Y., Yuan L.X., Lim T.K., Liu M. (2015). Haem-activated promiscuous targeting of artemisinin in Plasmodium falciparum. Nat. Commun..

[25] Yu L., Chen J.F., Shuai X., Xu Y., Ding Y., Zhang J., Yang W., Liang X., Su D., Yan C. (2016). Artesunate protects pancreatic beta cells against cytokine-induced damage via SIRT1 inhibiting NF-kappaB activation. J. Endocrinol. Investig..

[26] Heller L.E., Roepe P.D. (2019). Artemisinin-Based Antimalarial Drug Therapy: Molecular Pharmacology and Evolving Resistance. Trop. Med. Infect. Dis..

[27] Efferth T., Sauerbrey A., Olbrich A., Gebhart E., Rauch P., Weber H.O., Hengstler J.G., Halatsch M.E., Volm M., Tew K.D. (2003). Molecular modes of action of artesunate in tumor cell lines. Mol. Pharm..

[28] Efferth T., Dunstan H., Sauerbrey A., Miyachi H., Chitambar C.R. (2001). The anti-malarial artesunate is also active against cancer. Int. J. Oncol..

[29] Jiang F., Zhou J.-Y., Zhang D., Liu M.-H., Chen Y.-G. (2018). Artesunate induces apoptosis and autophagy in HCT116 colon cancer cells, and autophagy inhibition enhances the artesunate-induced apoptosis. Int. J. Mol. Med..

[30] Våtsveen T.K., Myhre M.R., Steen C.B., Wälchli S., Lingjærde O.C., Bai B., Dillard P., Theodossiou T.A., Holien T., Sundan A. (2018). Artesunate shows potent anti-tumor activity in B-cell lymphoma. J. Hematol. Oncol..

[31] Zhou C., Pan W., Wang X.P., Chen T.S. (2012). Artesunate induces apoptosis via a Bak-mediated caspase-independent intrinsic pathway in human lung adenocarcinoma cells. J. Cell. Physiol..

[32] Wang N., Zeng G.-Z., Yin J.-L., Bian Z.-X. (2019). Artesunate activates the ATF4-CHOP-CHAC1 pathway and affects ferroptosis in Burkitt’s Lymphoma. Biochem. Biophys. Res. Commun..

[33] Eling N., Reuter L., Hazin J., Hamacher-Brady A., Brady N.R. (2015). Identification of artesunate as a specific activator of ferroptosis in pancreatic cancer cells. Oncoscience.

[34] Roh J.-L., Kim E.H., Jang H., Shin D. (2017). Nrf2 inhibition reverses the resistance of cisplatin-resistant head and neck cancer cells to artesunate-induced ferroptosis. Redox Biol..

[35] Youns M., Hoheisel J.D., Efferth T. (2010). Traditional Chinese medicines (TCMs) for molecular targeted therapies of tumours. Curr. Drug Discov. Technol..

[36] Efferth T., Romero M.R., Wolf D.G., Stamminger T., Marin J.J., Marschall M. (2008). The antiviral activities of artemisinin and artesunate. Clin. Infect. Dis..

[37] Houben R., Angermeyer S., Haferkamp S., Aue A., Goebeler M., Schrama D., Hesbacher S. (2015). Characterization of functional domains in the Merkel cell polyoma virus Large T antigen. Int. J. Cancer.

[38] Hesbacher S., Pfitzer L., Wiedorfer K., Angermeyer S., Borst A., Haferkamp S., Scholz C.J., Wobser M., Schrama D., Houben R. (2016). RB1 is the crucial target of the Merkel cell polyomavirus Large T antigen in Merkel cell carcinoma cells. Oncotarget.

[39] Valeriote F., van Putten L. (1975). Proliferation-dependent cytotoxicity of anticancer agents: A review. Cancer Res..

[40] Compton M.M. (1992). A biochemical hallmark of apoptosis: Internucleosomal degradation of the genome. Cancer Metastasis Rev..

[41] Efferth T., Giaisi M., Merling A., Krammer P.H., Li-Weber M. (2007). Artesunate induces ROS-mediated apoptosis in doxorubicin-resistant T leukemia cells. PLoS ONE.

[42] Galluzzi L., Vitale I., Abrams J.M., Alnemri E.S., Baehrecke E.H., Blagosklonny M.V., Dawson T.M., Dawson V.L., El-Deiry W.S., Fulda S. (2012). Molecular definitions of cell death subroutines: Recommendations of the Nomenclature Committee on Cell Death 2012. Cell Death Differ..

[43] Bortner C.D., Cidlowski J.A. (2003). Uncoupling cell shrinkage from apoptosis reveals that Na+ influx is required for volume loss during programmed cell death. J. Biol. Chem..

[44] Tang D., Kang R., Berghe T.V., Vandenabeele P., Kroemer G. (2019). The molecular machinery of regulated cell death. Cell Res..

[45] Yan N., Zhang J.-J. (2019). The Emerging Roles of Ferroptosis in Vascular Cognitive Impairment. Front. Neurosci..

[46] Stockwell B.R., Friedmann Angeli J.P., Bayir H., Bush A.I., Conrad M., Dixon S.J., Fulda S., Gascón S., Hatzios S.K., Kagan V.E. (2017). Ferroptosis: A Regulated Cell Death Nexus Linking Metabolism, Redox Biology, and Disease. Cell.

[47] Dixon S.J., Lemberg K.M., Lamprecht M.R., Skouta R., Zaitsev E.M., Gleason C.E., Patel D.N., Bauer A.J., Cantley A.M., Yang W.S. (2012). Ferroptosis: An iron-dependent form of nonapoptotic cell death. Cell.

[48] Shacka J.J., Klocke B.J., Roth K.A. (2006). Autophagy, bafilomycin and cell death: The “a-B-cs” of plecomacrolide-induced neuroprotection. Autophagy.

[49] Xie Z., Xie Y., Xu Y., Zhou H., Xu W., Dong Q. (2014). Bafilomycin A1 inhibits autophagy and induces apoptosis in MG63 osteosarcoma cells. Mol. Med. Rep..

[50] Gao M., Monian P., Pan Q., Zhang W., Xiang J., Jiang X. (2016). Ferroptosis is an autophagic cell death process. Cell Res..

[51] Zhou B., Liu J., Kang R., Klionsky D.J., Kroemer G., Tang D. (2019). Ferroptosis is a type of autophagy-dependent cell death. Semin. Cancer Biol..

[52] Sato M., Kusumi R., Hamashima S., Kobayashi S., Sasaki S., Komiyama Y., Izumikawa T., Conrad M., Bannai S., Sato H. (2018). The ferroptosis inducer erastin irreversibly inhibits system x(c)- and synergizes with cisplatin to increase cisplatin’s cytotoxicity in cancer cells. Sci. Rep..

[53] Dixon S.J., Patel D.N., Welsch M., Skouta R., Lee E.D., Hayano M., Thomas A.G., Gleason C.E., Tatonetti N.P., Slusher B.S. (2014). Pharmacological inhibition of cystine-glutamate exchange induces endoplasmic reticulum stress and ferroptosis. Elife.

[54] Takahashi M., Nagai T., Okamura N., Takahashi H., Okano A. (2002). Promoting effect of beta-mercaptoethanol on in vitro development under oxidative stress and cystine uptake of bovine embryos. Biol. Reprod..

[55] Doll S., Proneth B., Tyurina Y.Y., Panzilius E., Kobayashi S., Ingold I., Irmler M., Beckers J., Aichler M., Walch A. (2017). ACSL4 dictates ferroptosis sensitivity by shaping cellular lipid composition. Nat. Chem. Biol..

[56] Yuan H., Li X., Zhang X., Kang R., Tang D. (2016). Identification of ACSL4 as a biomarker and contributor of ferroptosis. Biochem. Biophys. Res. Commun..

[57] Adam C., Baeurle A., Brodsky J.L., Wipf P., Schrama D., Becker J.C., Houben R. (2014). The HSP70 modulator MAL3-101 inhibits Merkel cell carcinoma. PLoS ONE.

[58] Serafin M.B., Bottega A., da Rosa T.F., Machado C.S., Foletto V.S., Coelho S.S., da Mota A.D., Horner R. (2019). Drug Repositioning in Oncology. Am. J. Ther..

[59] Tiwari M.K., Chaudhary S. (2020). Artemisinin-derived antimalarial endoperoxides from bench-side to bed-side: Chronological advancements and future challenges. Med. Res. Rev..

[60] Awad Adeel A. (2012). Time to switch from quinine. Sudan. J. Paediatr..

[61] Nass J., Efferth T. (2018). The activity of Artemisia spp. and their constituents against Trypanosomiasis. Phytomed. Int. J. Phytother. Phytopharm..

[62] Chen K., Shou L.M., Lin F., Duan W.M., Wu M.Y., Xie X., Xie Y.F., Li W., Tao M. (2014). Artesunate induces G2/M cell cycle arrest through autophagy induction in breast cancer cells. Anticancer Drugs.

[63] Li Y., Shan N.N., Sui X.H. (2020). Research Progress on Artemisinin and Its Derivatives against Hematological Malignancies. Chin. J. Integr. Med..

[64] Sharma B.N., Marschall M., Henriksen S., Rinaldo C.H. (2014). Antiviral effects of artesunate on polyomavirus BK replication in primary human kidney cells. Antimicrob. Agents Chemother..

[65] Sharma B.N., Marschall M., Rinaldo C.H. (2014). Antiviral effects of artesunate on JC polyomavirus replication in COS-7 cells. Antimicrob. Agents Chemother..

[66] Disbrow G.L., Baege A.C., Kierpiec K.A., Yuan H., Centeno J.A., Thibodeaux C.A., Hartmann D., Schlegel R. (2005). Dihydroartemisinin is cytotoxic to papillomavirus-expressing epithelial cells in vitro and in vivo. Cancer Res..

[67] Proneth B., Conrad M. (2019). Ferroptosis and necroinflammation, a yet poorly explored link. Cell Death Differ..

[68] Mou Y., Wang J., Wu J., He D., Zhang C., Duan C., Li B. (2019). Ferroptosis, a new form of cell death: Opportunities and challenges in cancer. J. Hematol. Oncol..

[69] Krishna S., Ganapathi S., Ster I.C., Saeed M.E.M., Cowan M., Finlayson C., Kovacsevics H., Jansen H., Kremsner P.G., Efferth T. (2014). A Randomised, Double Blind, Placebo-Controlled Pilot Study of Oral Artesunate Therapy for Colorectal Cancer. EBioMedicine.

[70] Deeken J.F., Wang H., Hartley M., Cheema A.K., Smaglo B., Hwang J.J., He A.R., Weiner L.M., Marshall J.L., Giaccone G. (2018). A phase I study of intravenous artesunate in patients with advanced solid tumor malignancies. Cancer Chemother. Pharmacol..

[71] von Hagens C., Walter-Sack I., Goeckenjan M., Storch-Hagenlocher B., Sertel S., Elsässer M., Remppis B.A., Munzinger J., Edler L., Efferth T. (2019). Long-term add-on therapy (compassionate use) with oral artesunate in patients with metastatic breast cancer after participating in a phase I study (ARTIC M33/2). Phytomed. Int. J. Phytother. Phytopharm..

[72] König M., von Hagens C., Hoth S., Baumann I., Walter-Sack I., Edler L., Sertel S. (2016). Investigation of ototoxicity of artesunate as add-on therapy in patients with metastatic or locally advanced breast cancer: New audiological results from a prospective, open, uncontrolled, monocentric phase I study. Cancer Chemother. Pharmacol..

[73] Rosen S.T., Gould V.E., Salwen H.R., Herst C.V., Le Beau M.M., Lee I., Bauer K., Marder R.J., Andersen R., Kies M.S. (1987). Establishment and characterization of a neuroendocrine skin carcinoma cell line. Lab. Investig..

[74] Van Gele M., Leonard J.H., Van R.N., Van L.H., Van B.S., Cocquyt V., Salwen H., De P.A., Speleman F. (2002). Combined karyotyping, CGH and M-FISH analysis allows detailed characterization of unidentified chromosomal rearrangements in Merkel cell carcinoma. Int. J. Cancer.

[75] Guastafierro A., Feng H., Thant M., Kirkwood J.M., Chang Y., Moore P.S., Shuda M. (2013). Characterization of an early passage Merkel cell polyomavirus-positive Merkel cell carcinoma cell line, MS-1, and its growth in NOD scid gamma mice. J. Virol. Methods.

