# Supplementary material for: Artesunate Affects T Antigen Expression and Survival of Virus-Positive Merkel Cell Carcinoma
Source: Cancers (Basel). 2020 Apr 9;12(4):919. doi: 10.3390/cancers12040919 (PMC7225937; doi:10.3390/cancers12040919)
Supplement: Supplementary file 1 [file cancers-12-00919-s001.pdf]

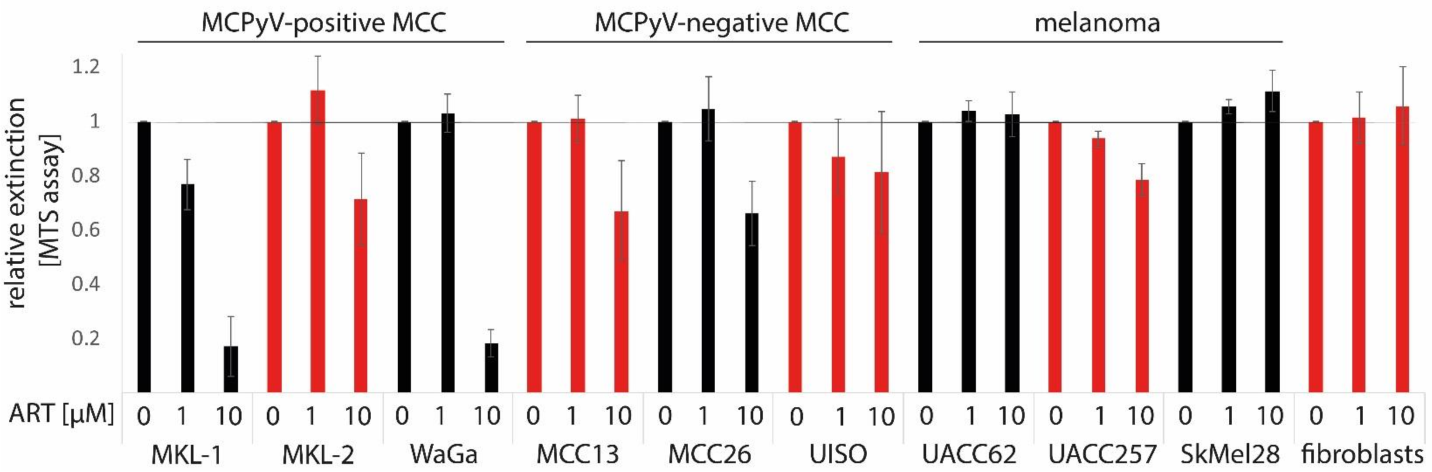

Supplementary Figure S1: MCC cell lines are more sensitive towards artesunate than melanoma cell lines or primary fibroblasts. The indicated cells were seeded in sixtuplicates (fibroblasts values represent means of three individual preparations) in 96-well plates and were cultured for 5 days in the absence or presence of the indicated artesunate concentrations. Following incubation with the MTS reagent absorbance at 490 nm was determined, and values normalized to untreated controls are depicted.

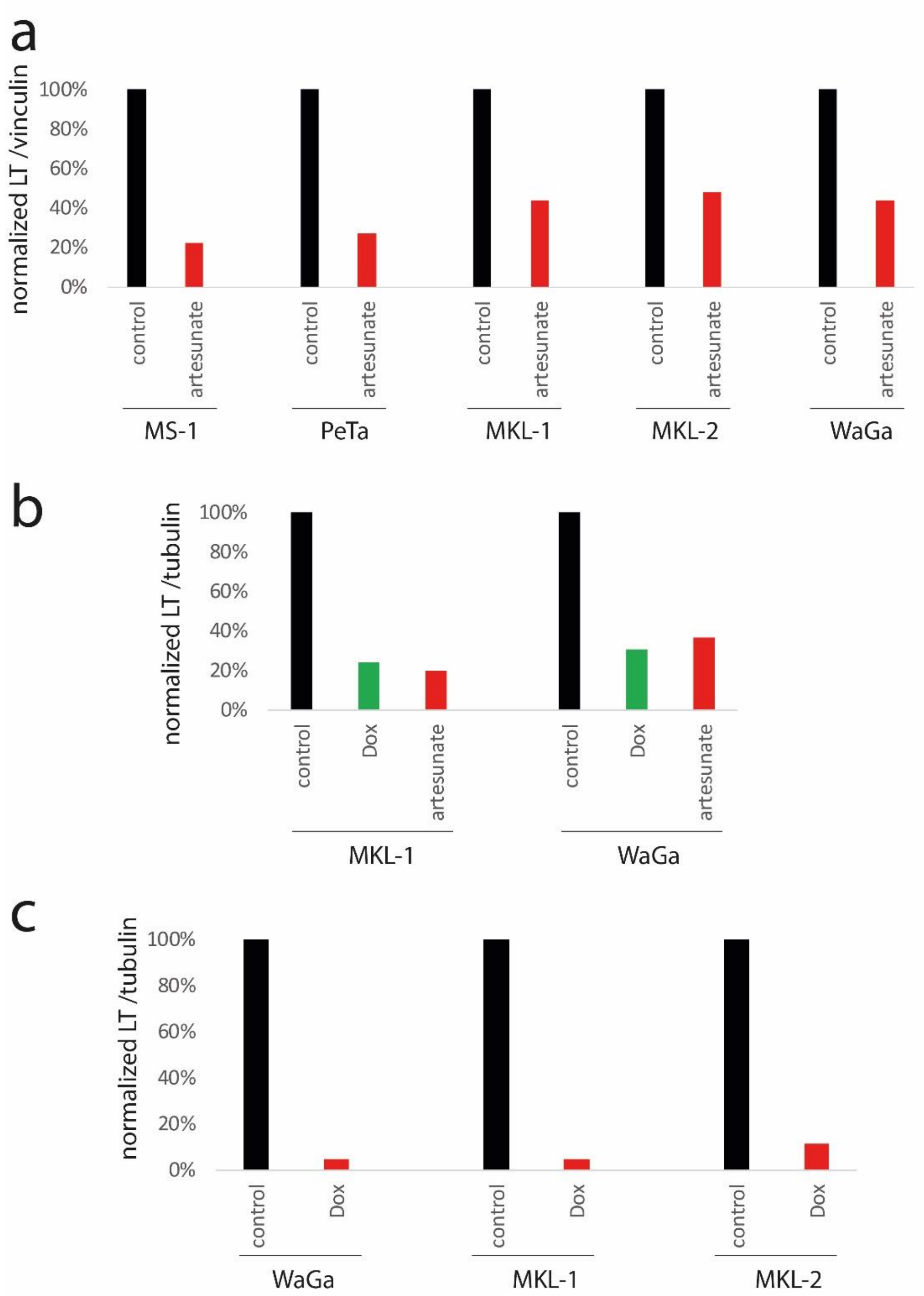

Supplementary Figure S2: Densitometric analyses of all immunoblots presented in the publication. ImageJ was used to quantify intensity of LT and loading controls (tubulin or vinculin) signals. LT values are presented relative to the loading control and normalized to untreated cells. Quantification of blots presented in Fig 1a (**a**), Fig. 1c (**b**) or Fig. 2 (**c**).

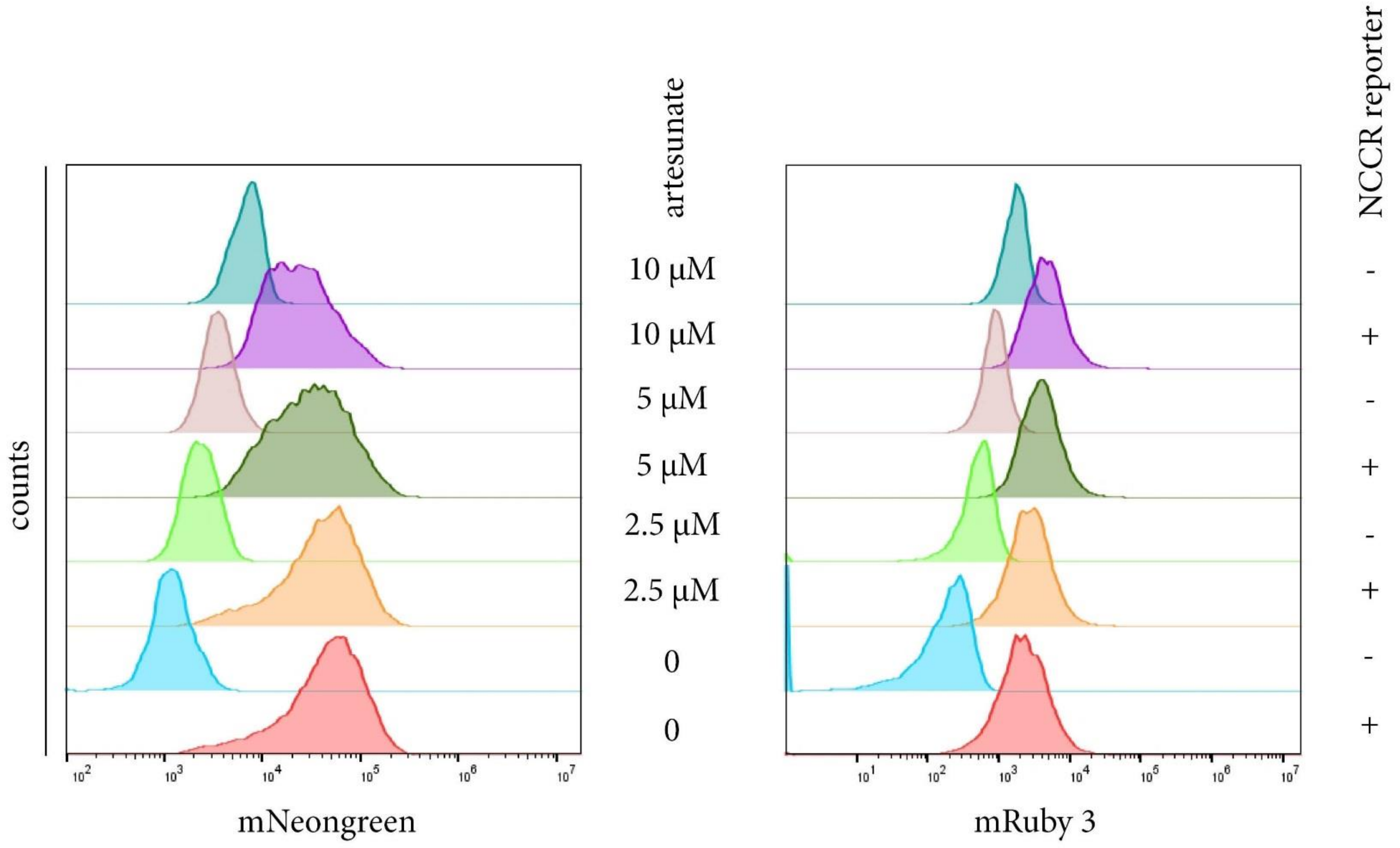

Supplementary Figure S3: Artesunate represses NCCR driven early region transcription (representative histograms of the NCCR Reporter-Assay corresponding to the bar graph in Figure 1b). MKL-1 cells stably transduced with a bi-directional NCCR reporter construct (see Supplementary Figure S6) were treated for five days with the indicated artesunate concentrations followed by flow cytometric analysis. mNeongreen and mRuby3 Fluorescence representing early and late region, respectively, were recorded. MKL-1 cells treated with the same artesunate concentrations served as controls for background subtraction.

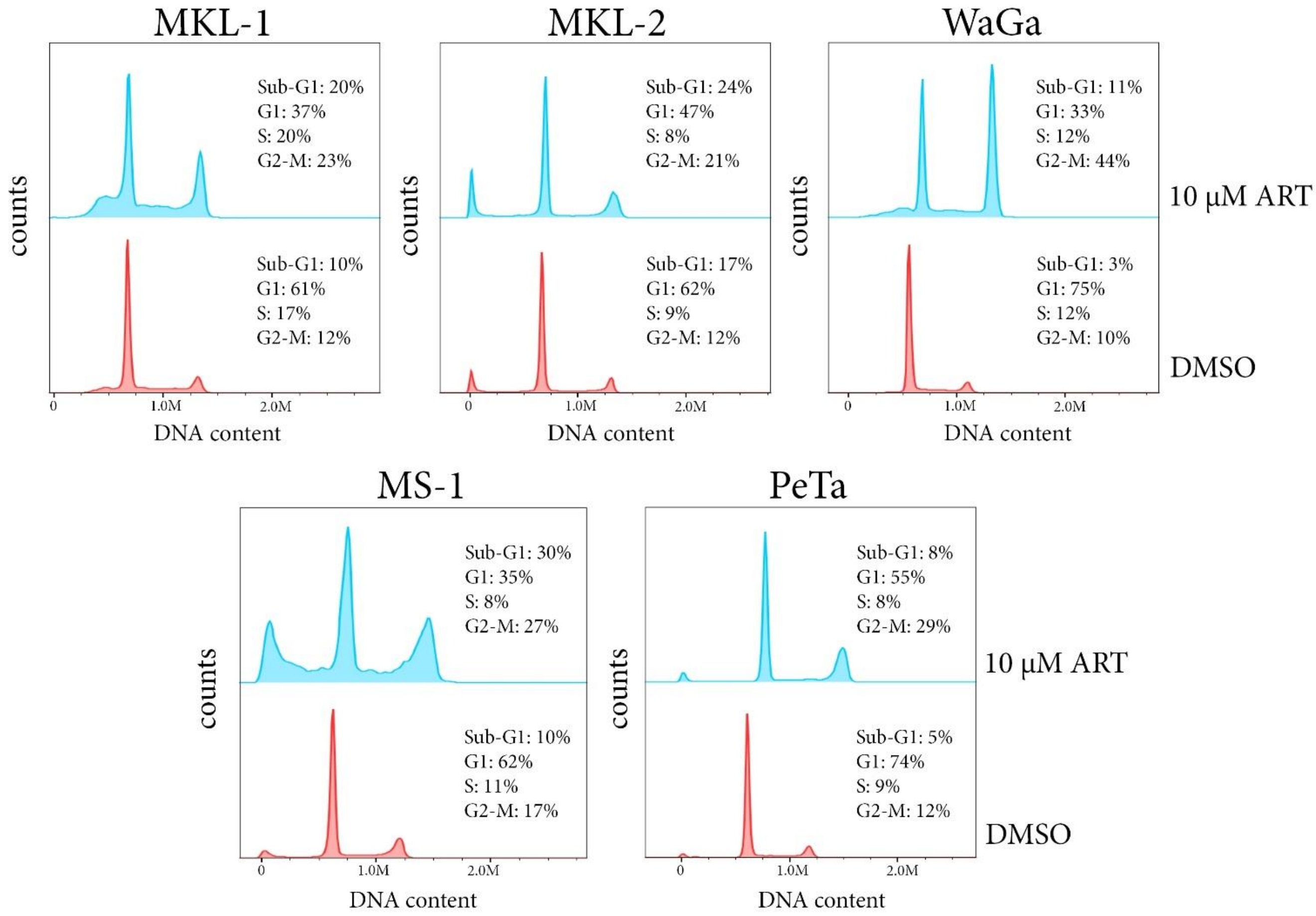

Supplementary Fig. S4: Artesunate induces G2/M arrest in MCPyV-positive MCC cells. The indicated MCC cell lines were incubated in the absence or presence of artesunate (10 µM). After two days, cells were fixed and stained with propidium iodide followed by flow cytometry. The percentage of cells in sub-G1 and the different cell cycle phases are indicated.

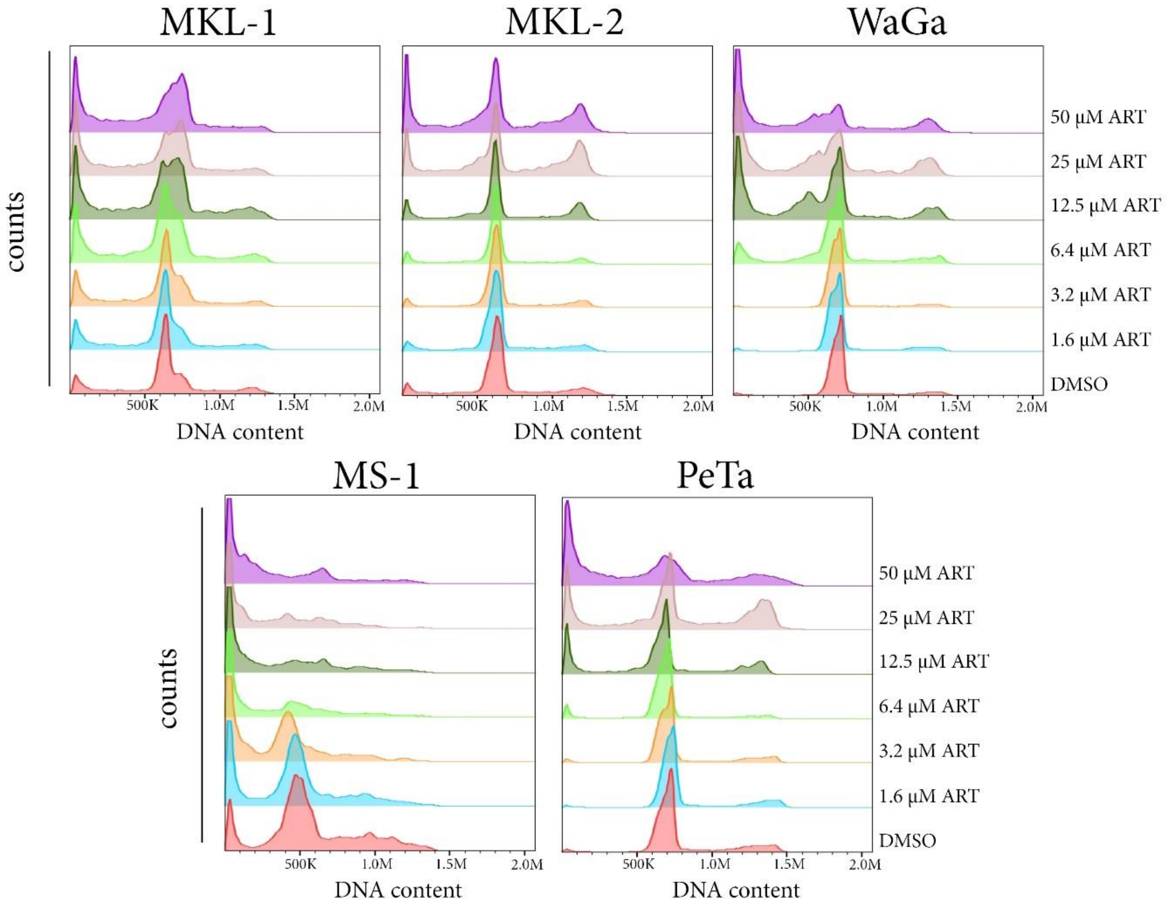

Supplementary Fig. S5: Propidium iodide staining of artesunate treated MCPyV-positive MCC cells (representative histograms corresponding to Figure 3a). The indicated MCC cell lines were treated with different concentrations of artesunate. After four days, cells were fixed and stained with propidium iodide followed by flow cytometry.

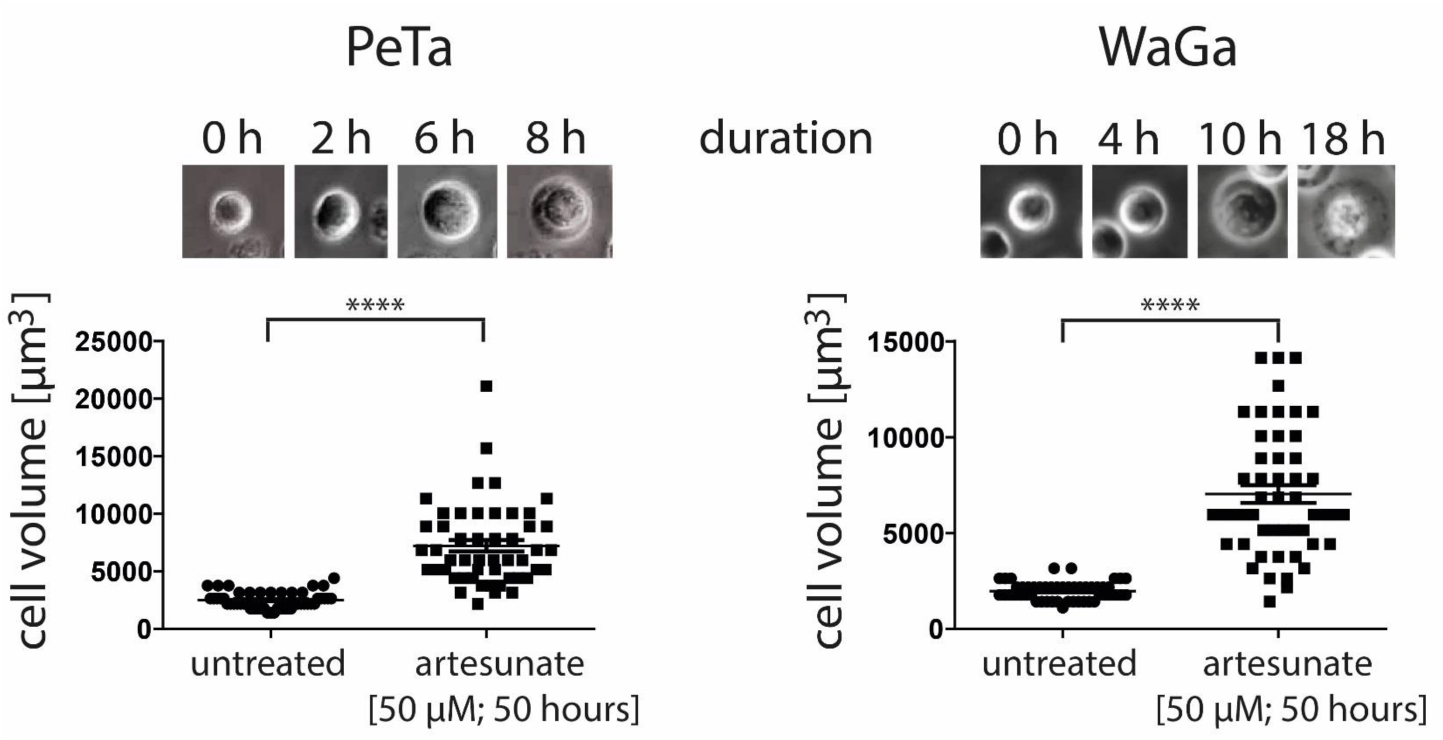

Supplementary Figure S6: Artesunate induces cell swelling of MCPyV-positive MCC cells. Time lapse microscopy was used to analyze morphological changes of the suspension cell lines PeTa and WaGa upon incubation with 50 µM artesunate. In the upper part examples of the changes observed in the course of time of individual cells are given. The cellular volume of 50 cells prior and after 50 hours artesunate treatment was estimated, and the values are presented in the lower part of the figure. The means were compared using the Mann-Whitney test (****$p < 0.0001$) since data were not normally distributed.

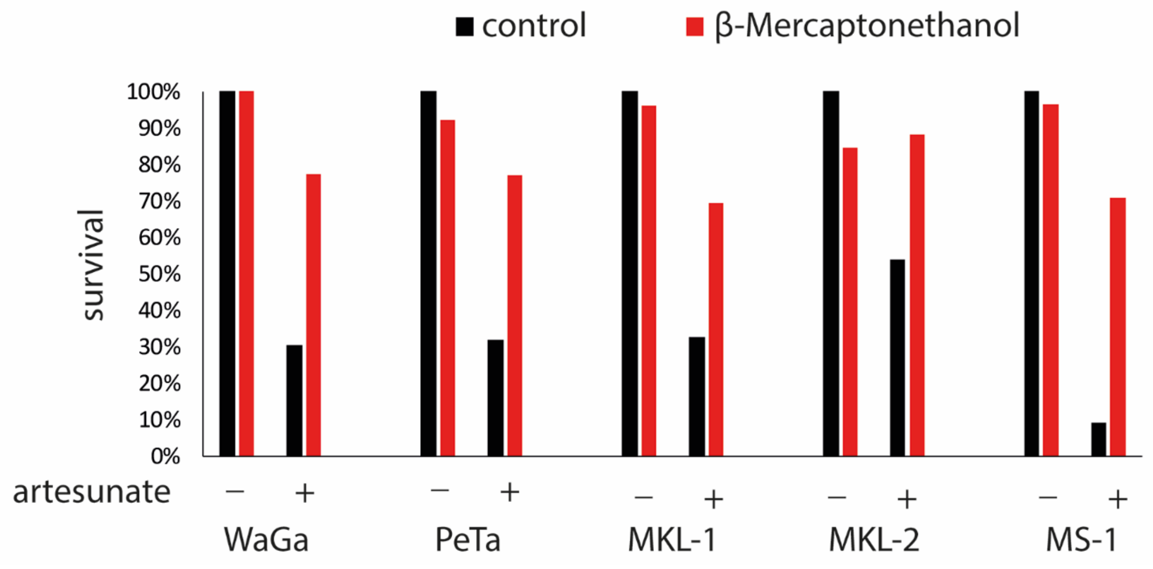

Supplementary Figure S7: β-Mercaptoethanol, an activator of cystine uptake, represses artesunate-induced cell death. The indicated MCPyV-positive MCC cell lines were cultivated in the absence or presence of artesunate (50 µM). Additionally, 66 µM β-mercaptoethanol were applied. After three days viability was analyzed using the trypan blue exclusion assay. Values are normalized to the untreated controls.

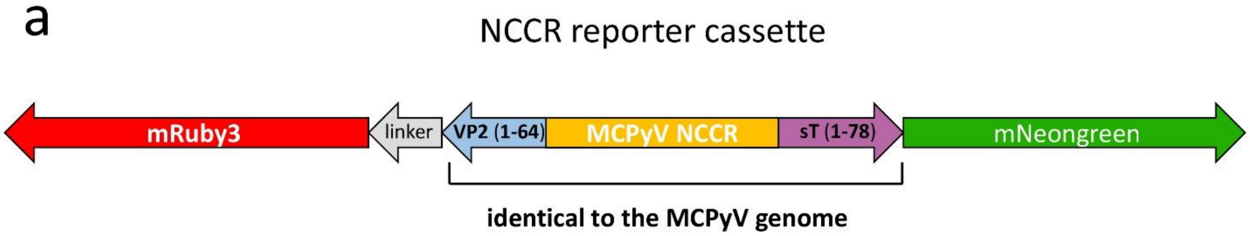

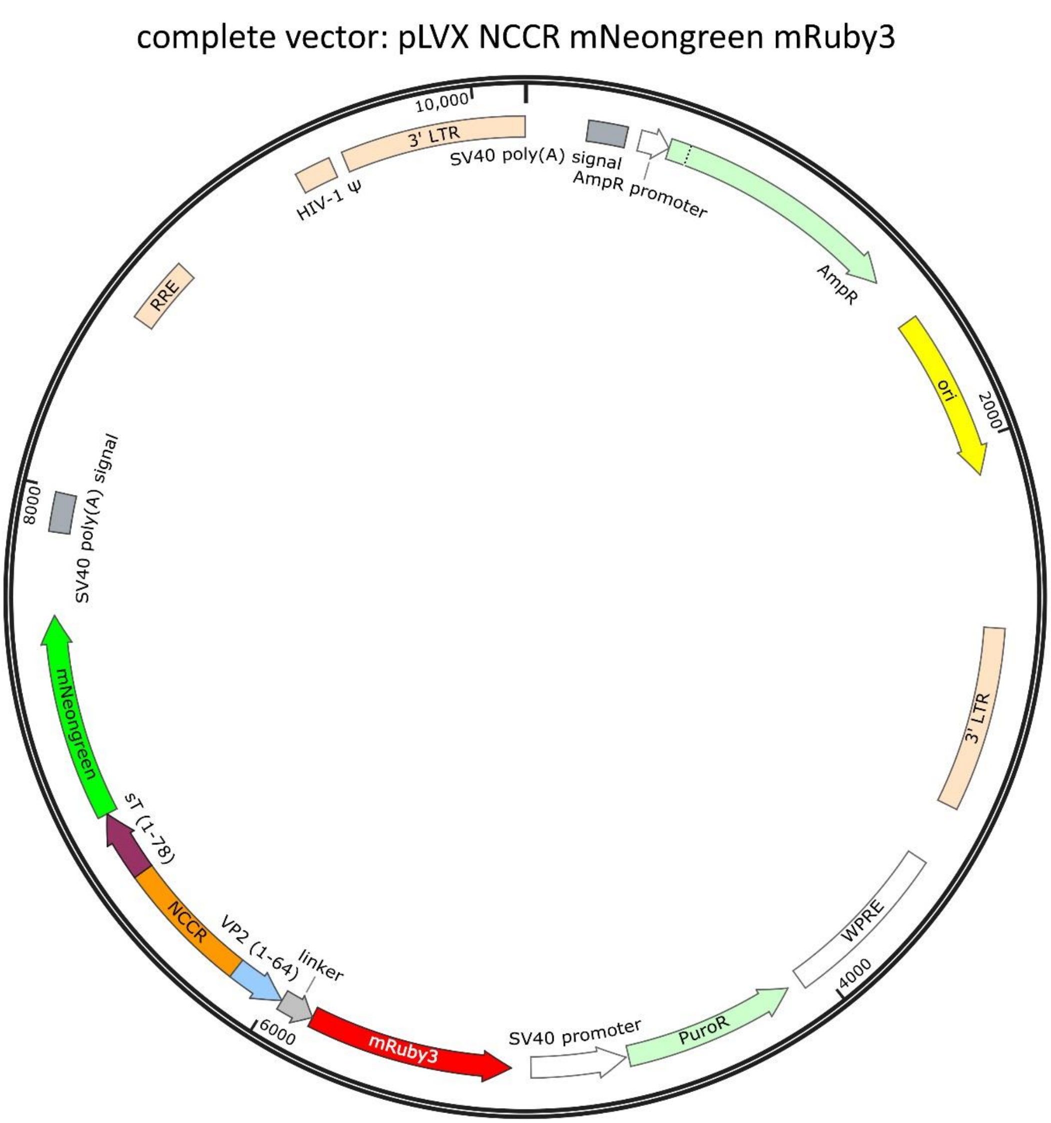

Supplementary Figure S8: An MCPyV NCCR reporter construct. **a)** Graphical display of the reporter cassette containing the central bi-directional MCPyV NCCR plus coding sequences for the N-terminus of sT and VP2 as well as in frame cloned mNeongreen and mRuby. **b)** The complete lentiviral vector with the integrated NCCR reporter cassette is depicted. The Snapgene file is available upon request.

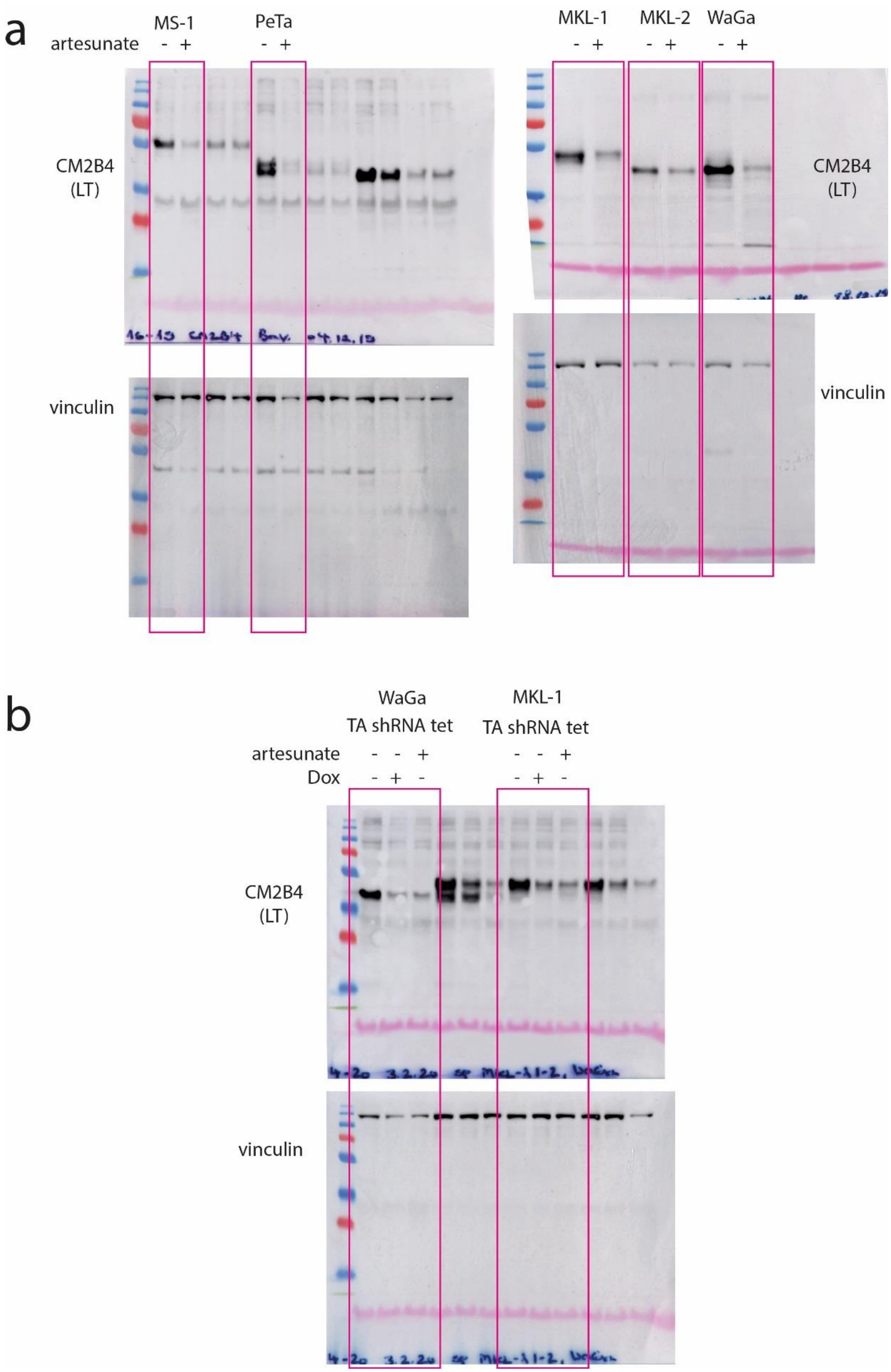

Supplementary Fig. S9: Uncropped blots from Figure 1a (a) and 1c (b).

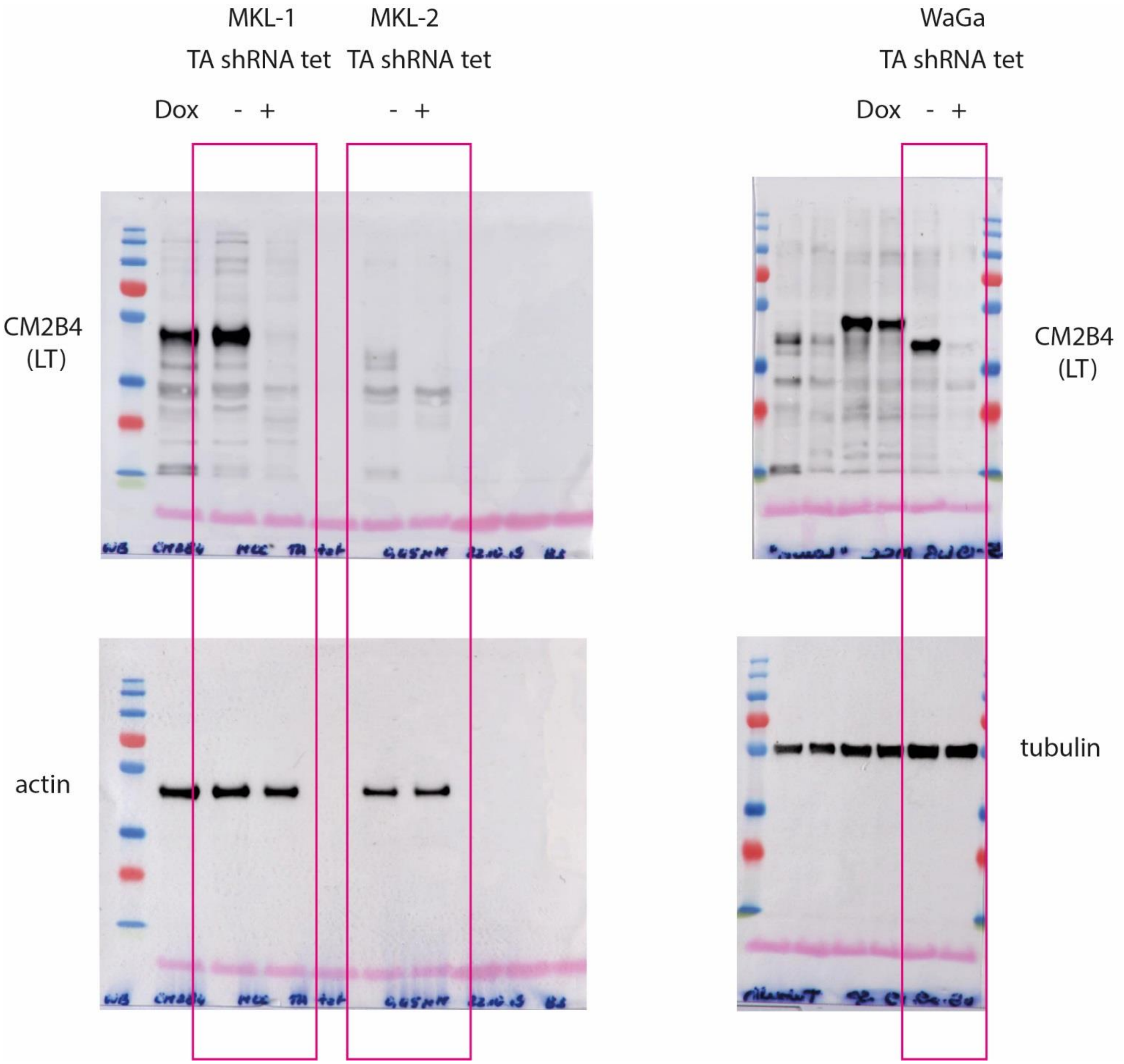

Supplementary Figure S10: Uncropped blots from Figure 2.
